# Inhibition of endocytosis suppresses the nitric oxide-dependent release of Cl^-^ in retinal amacrine cells

**DOI:** 10.1371/journal.pone.0201184

**Published:** 2018-07-25

**Authors:** Vernon K. Dunn, Evanna Gleason

**Affiliations:** Department of Biological Sciences, Louisiana State University, Baton Rouge, Louisiana, United States of America; Indiana University School of Medicine, UNITED STATES

## Abstract

Our lab has previously shown that nitric oxide (NO) can alter the synaptic response properties of amacrine cells by releasing Cl^-^ from internal acidic compartments. This alteration in the Cl^-^ gradient brings about a positive shift in the reversal potential of the GABA-gated current, which can convert inhibitory synapses into excitatory synapses. Recently, we have shown that the cystic fibrosis transmembrane regulator (CFTR) Cl^-^ channel is involved in the Cl^-^ release. Here, we test the hypothesis that (acidic) synaptic vesicles are a source of NO-releasable Cl^-^ in chick retinal amacrine cells. If SVs are a source of Cl^-^, then depleting synaptic vesicles should decrease the nitric oxide-dependent shift in the reversal potential of the GABA-gated current. The efficacy of four inhibitors of dynamin (dynasore, Dyngo 4a, Dynole 34–2, and MiTMAB) were evaluated. In order to deplete synaptic vesicles, voltage-steps were used to activate V-gated Ca^2+^ channels and stimulate the synaptic vesicle cycle either under control conditions or after treatment with the dynamin inhibitors. Voltage-ramps were used to measure the NO-dependent shift in the reversal potential of the GABA-gated currents under both conditions. Our results reveal that activating the synaptic vesicle cycle in the presence of dynasore or Dyngo 4a blocked the NO-dependent shift in E_GABA_. However, we also discovered that some dynamin inhibitors reduced Ca^2+^ signaling and L-type Ca^2+^ currents. Conversely, dynasore also increased neurotransmitter release at autaptic sites. To further resolve the mechanism underlying the inhibition of the NO-dependent shift in the reversal potential for the GABA-gated currents, we also tested the effects of the clathrin assembly inhibitor Pitstop 2 and found that this compound also inhibited the shift. These data provide evidence that dynamin inhibitors have multiple effects on amacrine cell synaptic transmission. These data also suggest that inhibition of endocytosis disrupts the ability of NO to elicit Cl^-^ release from internal stores which may in part be due to depletion of synaptic vesicles.

## Introduction

The transmission of information through neuronal circuits depends on the function and regulation of synapses. Chemical synapses are especially important because the diversity of physiological types of synapses and their flexibility can alter the conveyance of information. In the vertebrate retina, Amacrine cells (ACs) form primarily gamma-aminobutyric acid (GABA)-ergic and glycinergic synapses with ganglion cells, bipolar cells, and other ACs. GABA and glycine bind to ionotropic postsynaptic receptors and activate Cl^-^ channels. The excitatory or inhibitory effect of the postsynaptic response to these neurotransmitters is dependent upon the electrochemical gradient for Cl^-^ across the postsynaptic plasma membrane. Thus, understanding the regulation of cytosolic Cl^-^ at synapses is fundamental to understanding the full flexibility of neuronal circuitry.

We have previously shown that nitric oxide (NO), can alter the response properties of postsynaptic ACs that express Cl^-^ conducting GABA_A_ receptors. Specifically, a novel mechanism involving a NO-dependent release of Cl^-^ (NOdrCl) from internal stores into the cytoplasm has been demonstrated [1]. This release brings about a positive shift in the equilibrium potential for Cl^-^, which can convert these GABAergic and inhibitory synapses into excitatory synapses [1]. Endosomes are strong candidates for contributing to NO-releasable Cl^-^ because they contain millimolar [2] concentrations of Cl^-^. Synaptic vesicles (SVs), derived from early endosomes, have an estimated Cl^-^ concentration of between 40–50 mM [3]. Additionally, our lab has provided evidence that Cl^-^ is coming from acidic organelles, and that intact endosomal proton gradients are required for the NOdrCl [4]. Acidification of endosomes occurs via the simultaneous proton pumping of the V-ATPase and charge compensation by the counter ion Cl^-^, which is moved into the cytosol via an as yet unknown Cl^-^ transport mechanism. The cystic fibrosis transmembrane conductance regulator (CFTR) is a Cl^-^ transporter that can be found in internal membranes of vertebrate neurons [5, 6]. Recently our lab has shown that pharmacological inhibition and knockdown of CFTR expression in ACs blocks the NO-dependent shift in E_rev_-_GABA_ [7].

Here we aim to test the hypothesis that SVs can release Cl^-^ and contribute to the NOdrCl. Nitric oxide synthase expression is found in subsets of amacrine cells in both mammalian [8–17] and avian retinas [18, 19] and has been localized specifically to amacrine cell presynaptic terminals at the EM level in the turtle retina [20]. Furthermore, amacrine cells participate in reciprocal synapses with other amacrine cells where pre and postsynaptic elements within the same process can exist side by side [18, 19, 21–24]. As such, Cl^-^ released from SVs can influence the postsynaptic responses at adjacent synaptic sites. To uncover the role of SVs in the NOdrCl, we made whole cell voltage-clamp recordings of cultured ACs. After 8 days in culture, ACs form functional GABAergic synapses with each other similar to GABAergic synapses in the intact retina [25, 26]. This simplified system allows for the isolation of GABAergic synapses and the elimination of synapses from other cell types in the intact retina. Our objective was to deplete the SV pool by driving exocytosis with depolarizing voltage steps while inhibiting compensatory endocytosis pharmacologically. Dynamin is a key player in endocytosis functioning as a GTP-dependent pinchase, driving fission of endocytosing vesicles. In mammals, there are three isoforms of dynamin (dynamin 1–3). Dynamin 1 is expressed at high levels in neurons [27]. Dynamin 2 is expressed ubiquitously [28], and dynamin 3 is almost exclusively expressed in the brain and testis [29]. There is also evidence that all three isoforms are expressed in the developing rat brain [30, 31]. In the chicken, full predicted sequences for dynamin 1 and 3 have been identified while only a partial predicted sequence is available for dynamin 2. However, dynamin 1 and 3 are highly conserved between chicken and rodents (dynamin 1 rat 96%, mouse 94%; dynamin 3 rat 92%, mouse 93%; scores are for identity). All three isoforms have the four domains of dynamin: a GTPase domain, a plextrin homology domain that interacts with membrane lipids, a GTPase effector domain involved in assembly [32, 33], and a proline-rich domain [34–36]. Dynasore interferes with the GTPase activity of dynamin during endocytosis [37]. The more potent dynasore analogs Dyngo 4a [38] and Dynole 34–2 [39] also target the GTPase activity of dynamin. MiTMAB targets the plekstrin homology domain of which interacts with membrane lipids and assists in vesicle fission [40].

Our findings reveal that the dynamin inhibitors suppressed endocytosis in ACs and two of them (dynasore and Dyngo-4a) interfered with the NOdrCl. However, we have also discovered that the dynamin inhibitors can alter Ca^2+^ signaling, voltage-gated Ca^2+^ currents and synaptic transmission. Interestingly, the clathrin inhibitor Pitstop 2 also reduced the NOdrCl suggesting that disruption of endocytosis is at least one factor involved in the inhibition of NOdrCl.

## Materials and methods

### Cell culture

The Louisiana State University Institutional Animal Care and Use Committee has granted a formal waiver of ethical approval. Eight-day chick embryo eyes were removed and the retinas dissociated and cultured as previously described [4]. Cell cultures were maintained under 5% CO_2_ atmosphere at 37°C. ACs that had been in culture for 9–13 days (embryonic equivalent days 17–21) were used for experiments because AC-AC GABAergic synapses are functional from embryonic equivalent day 16 onwards. ACs used for electrophysiology and live-cell imaging experiments were identified based on morphology as previously described [25].

### Electrophysiology

Culture dishes were mounted on the stage of an Olympus IX70 inverted microscope. A reference Ag/AgCl pellet electrode in 3M KCl grounded the dish via 1% agarose bridge containing 3M KCl. Patch pipettes were pulled from thick-walled borosilicate glass (O.D.:1.5 mm, I.D.:0.86 mm; Sutter Instruments, Novato, CA) using a P-97 Flaming/Brown puller (Sutter Instruments). Patch pipette resistances were between 4–8 MΩ when measured in the bath. Whole-cell recordings were performed using an Axopatch 1-D amplifier, Digidata 1322A data-acquisition board, and pClamp 10.0 software (Molecular Devices, Sunnyvale, CA). External solutions were continuously perfused (1.5–2 mL/min) using a pressurized 8-channel perfusion system (Automate Scientific, Berkeley, CA). Series resistance was measured but series resistance compensation was not employed. Using the pClamp 10.0 calculator, the junction potential was estimated to be 14.5 mV and 21.5 mV for ruptured patch and perforated patch solutions, respectively. Voltage ramp data for determining E_rev_-_GABA_ were analyzed by making corrections for series resistance error and junction potential then subtracting the leak current (also corrected) that was obtained from recording ramp currents in the absence of GABA. For autaptic recordings, the ACs were not in contact with other neurons, ruling out synaptic input from unclamped cells. Charge transfer at autapses was calculated as previously described and reported as pC [41]. Inward Ca^2+^ currents were often contaminated and obscured by outward synaptic current during the voltage step.

### Solutions and reagents

All reagents were purchased from Sigma-Aldrich, unless otherwise indicated. The external solution used for electrophysiology experiments contained (in mM): NaCl (116.7), KCl (5.3), Tetrathylammonium (TEA)-Cl (20), CaCl_2_ (6.0), MgCl_2_ (0.82), HEPES (10.0), glucose (5.6). Tetrodotoxin citrate (TTX) (300 μM, Abcam, Cambridge, UK) was added to external solutions to block voltage-gated Na^+^ channels. For GABA-gated current recordings, the voltage-gated Ca^2+^ channel blocker La^3+^ (50 μM) was added to the external solution. For recordings of voltage-gated Ca^2+^ currents, 10 μM bicuculline was added to the external solution to block GABA_A_ receptors. The pH of external solutions was adjusted to 7.4 using HCl.

Internal solutions for perforated patch recordings contained (in mM): Cs Methanosulfonate (135.0), CsCl (10.0), MgCl (4.0), CaCl_2_ (0.2), EGTA (1.1), HEPES (10.0), and NaCl (1.0) and Amphotericin B (Calbiochem, San Diego, CA). Amphotericin B stock (54 mM) was mixed and sonicated with Pluronic F-127 (2.5% w/v in DMSO, Life Technologies) in a 1:2 ratio. The Amphotericin B stock and pluronic mixture was vortexed and sonicated for 30 seconds each then added to 5 mL of internal B solution for a final concentration of 200 μg/mL and kept on ice during experiments. Internal solutions for ruptured patch recordings contained (in mM): Cs Methanosulfonate (100), CsCl (10.0), CaCl_2_ (0.1), MgCl_2_ (2.0), HEPES (10.0) and EGTA (1.1) in combination with an ATP regeneration system. The ATP regeneration system contained 50 lu/mL creatine phosphokinase, 20 mM creatine phosphate, 1 mM ATP disodium, 3 mM ATP dipotassium and 2 mM GTP. For experiments targeting dynamin GTPase activity, the GTPase inhibitor GTP*γ*S (Sigma-Aldrich, St. Louis, MO) replaced GTP in the internal solution at 4 mM. Internal solutions were filtered through a 0.2 μM Nalgene (Thermo Fischer Scientific, Waltham, MA). The pH for internal solutions was adjusted to 7.4 using CsOH.

Dynamin inhibitors were prepared as 100 mM stocks in DMSO. Stocks were added to external solutions, then mixed and sonicated for final concentrations of: dynasore (80 μM, TOCRIS, Bristol, UK), MiTMAB (30 μM, Abcam), Dyngo 4a (30 μM, Abcam), and Dynole 34–2 (10 μM, Abcam). A DMSO-only external solution was used as a control. The actin polymerization inhibitors Cytochalasin D (Enzo, Farmingdale, NY) and Latrunculin B (Enzo) were mixed and sonicated with DMSO to make 100 mM stocks and added to the TEA external solution. The final concentration of both inhibitors was 2 μM. For experiments measuring autaptic currents, external solution with Li^+^ replacing Na^+^ was perfused just during collection of the autaptic data to remove the contaminating current arising from the Na-Ca exchanger. The time the cells were exposed to Li^+^ was kept as short as possible (4 sec) to minimize effects due to inhibiting exchanger function. Additionally, CsCl was increased to 145 mM (by replacing Cs methanesulfonate) in the internal solution in order to raise the equilibrium potential for Cl^-^ and increase the driving force on Cl^-^ when recording synaptic currents at -70 mV)

### NO solutions

NO solutions were prepared by bubbling Fisher double-distilled pure water with argon for 20 minutes followed by bubbling with pure NO gas for 5 minutes. The NO was then sealed and protected from light and stored at 4°C until use. NO (20–50 μL) was injected into the perfusion line and has been estimated to reach the recorded cell in ~3s and to remain for ~3-5s [1].

### Fluorescence imaging

The external solution used for imaging experiments contained (in mM): KCl (5.4), NaCl (136.9), CaCl_2_ (3), MgCl_2_ (1), HEPES (3.0), and Glucose (5.5). The lipophilic dye FM1-43 (4 μM, Life Technologies, Carlsbad, CA) was added to the imaging external solution. Culture dishes were mounted on an Olympus IX70 inverted microscope equipped with a Sensicam QE CCD camera (QImaging, Surrey, BC). After gaining electrical access, cells were exposed FM dye and after voltage steps were delivered, the dye was washed out for 1–2 minutes before continuing the experiment. All images were acquired with SlideBook 5.0 software (3I, Denver, CO) and analyzed using Graphpad software (La Jolla, CA).

For Ca^2+^ imaging experiments, the cell permeable Ca^2+^ indicator Oregon Green 488 Bapta-1 AM (OGB, Thermo Fisher Scientific) stocks (2 mM) were combined with Pluronic F-127(2.5% w/v in DMSO, Life Technologies) in 1:1 (μL) ratio, and sonicated. The 2 μL OGB and Pluronic F-127 mixture was added to 1 mL of Hank’s buffered salt solutions, vortexed, and sonicated for a final concentration of 2 μM. Cells were incubated in the dark at room temperature for 1 hour in the OGB/Pluronic/HBSS mixture then rinsed with normal imaging external solution. Images were captured every 3 seconds with an exposure time of 1 second. Data were gathered from regions of interest in cell bodies. A high K^+^ solution (50 mM) was perfused onto the cells for 6 seconds to depolarize the cells and activate voltage-gated Ca^2+^ channels in the absence or presence of a dynamin inhibitor. The effects of the inhibitors were independent of the order of presentation. Mean F/F_0_ in all Ca^2+^ imaging experiments was calculated by averaging the ROI mean fluorescence for the first 15 time points before exposure to K^+^ and then dividing all the other values by that number. Images and data acquisition were achieved using Slidebook 5.0 software.

### Immunocytochemistry

ACs were grown on glass coverslips for 8 days in culture and then fixed in 2% paraformaldehyde for 30 minutes at 4°C. Fixed cells were incubated for 30 minutes at 4°C in a blocking solution containing dilution solution (PBS, 1% bovine serum albumin, 0.5% saponin) with 5% normal goat serum. Primary polyclonal antibodies were raised against rabbit CFTR (Catalog no. ab131553; Abcam). We have previously characterized this antibody with a western blot showing a single band at the appropriate molecular weight [7]. Antibodies were diluted at 1:1000 in dilution solution and applied to cells at 4°C overnight then washed with PBS. Secondary goat anti-rabbit DyLight 488 antibody (no. 355552; Thermo Fisher Scientific) was diluted to 1:500 in dilution solution. Cells were incubated for 1 hour at room temperature in secondary antibodies then washed in PBS. Secondary antibody-only controls were prepared in the same solutions and produced no labeling [7]. The primary monoclonal antibody raised against SV2 developed by K.M. Buckley of Harvard Medical School was obtained from the Developmental Studies Hybridoma Bank, created by the NICHD of the NIH and maintained at The University of Iowa, Department of Biology, Iowa City, IA 52242.This antibody has been demonstrated to be effective in chicken tissue according to the product information from the Developmental Studies Hybridoma Bank. Supernatant was diluted at 1:2 and incubated at room temperature for 1 hour. Secondary goat anti-mouse Dylight 550 antibody (Thermo Fisher Scientific) was diluted to 1:1000 in dilution solution. Cells were incubated again at room temperature for 1 hour then washed in PBS. No non-specific labeling was observed in secondary-only controls. After washing, coverslips were mounted in Prolong Diamond Antifade mounting medium (Thermo Fisher Scientific). Cells were viewed on an Olympus IX70 microscope equipped with epifluorescence and images were acquired using Slidebook 5.0.

### Data analysis

Data from electrophysiology recordings were analyzed using Clampfit 10.0 software, and plotted in Origin 8.0 (OriginLab, Northampton, MA). Imaging data were analyzed using Slidebook and Origin and plotted in Origin. Figures were assembled in Adobe Illustrator (Adobe Systems, San Jose, CA). Data are reported as means ± SD. Statistical analyses were done in GraphPad Prism 6 (Graphpad Software, La Jolla, CA) using the paired and unpaired student’s *t*-test, and one-way analysis of variance [42], and Tukey honest significant difference post hoc as appropriate. Levels of significance are represented as *p < 0.05, **p < 0.01, ***p < 0.001, ****p< 0.0001.

## Results

### CFTR expression at putative synapses

**Fig 1:**
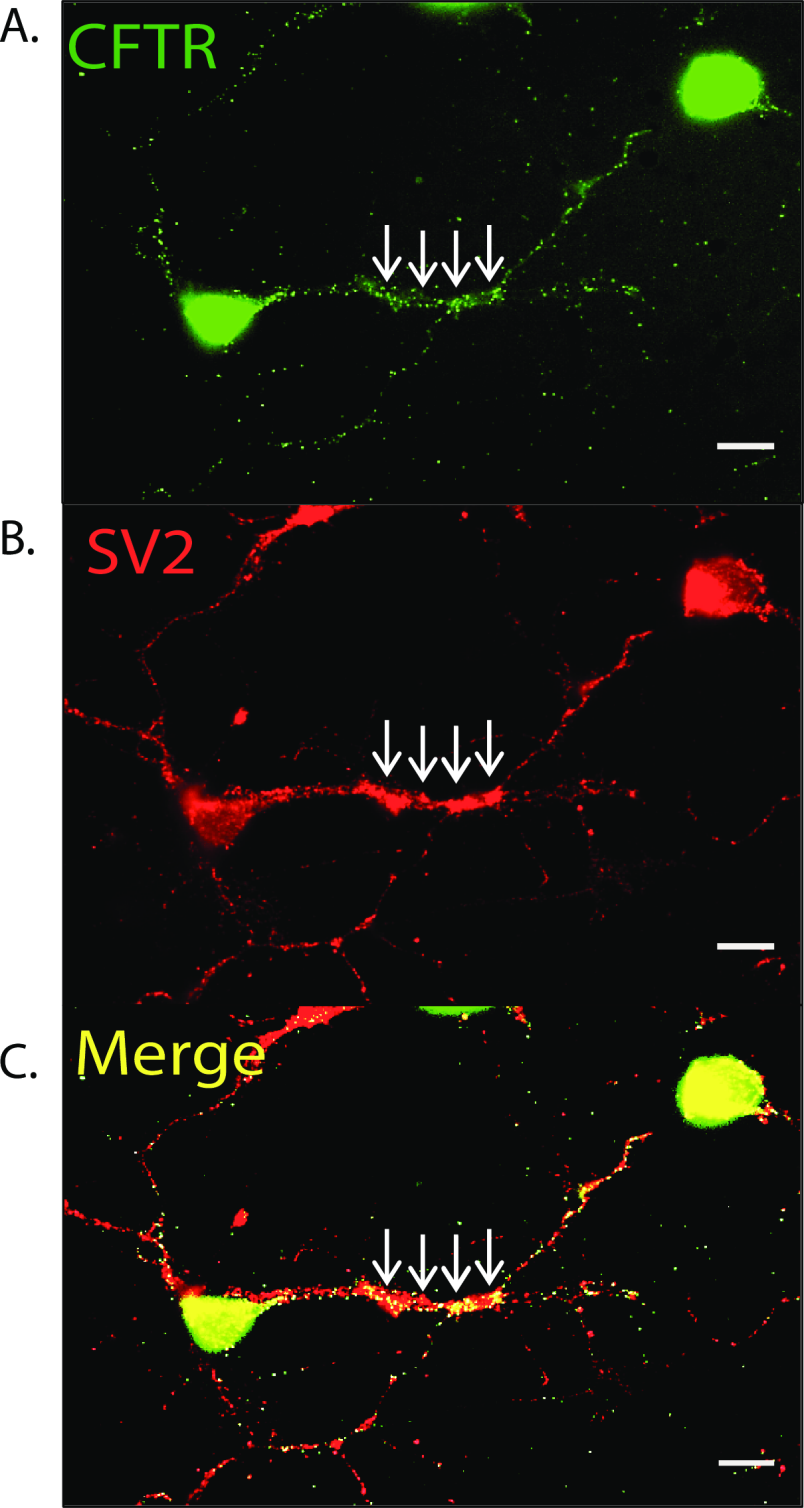
CFTR co-localizes with a synaptic vesicle protein. **A**, Polyclonal anti-CFTR antibody labels retinal amacrine cell bodies and processes. Arrows indicate a region where multiple AC processes are in contact, possibly a synaptic region (arrows). **B**, Anti-SV2 labeling is found throughout the cells but is especially strong in regions of process contact (arrows). **C**, Merged images of CFTR and SV2 labeling shows strong co-localization in the region of process contact. Scale bars are 10 μm.

We have demonstrated that CFTR expression and function is required for the NOdrCl [7]. We began here by testing whether the CFTR protein was localized to the same regions of the cell as SV2, a synaptic vesicle marker protein. In cultured ACs, the anti-CFTR antibody labeling overlapped with populations of vesicles labelled with SV2. Co-labelling was observed at sites of extensive process contact, which can indicate synaptic sites. (Fig 1). The Pearson’s R-value from 48 regions of interest in amacrine cell processes was 0.38 ± 15 (p<0.0001) indicating a positive correlation for the expression of CFTR and SV2. This co-localization suggests that CFTR might be expressed on SVs. These results are consistent with the possibility that NOdrCl via CFTR activity on SVs could contribute to the elevation in cytosolic Cl^-^.

### Activating voltage-gated Ca^2+^ channels promotes endocytosis

**Fig 2:**
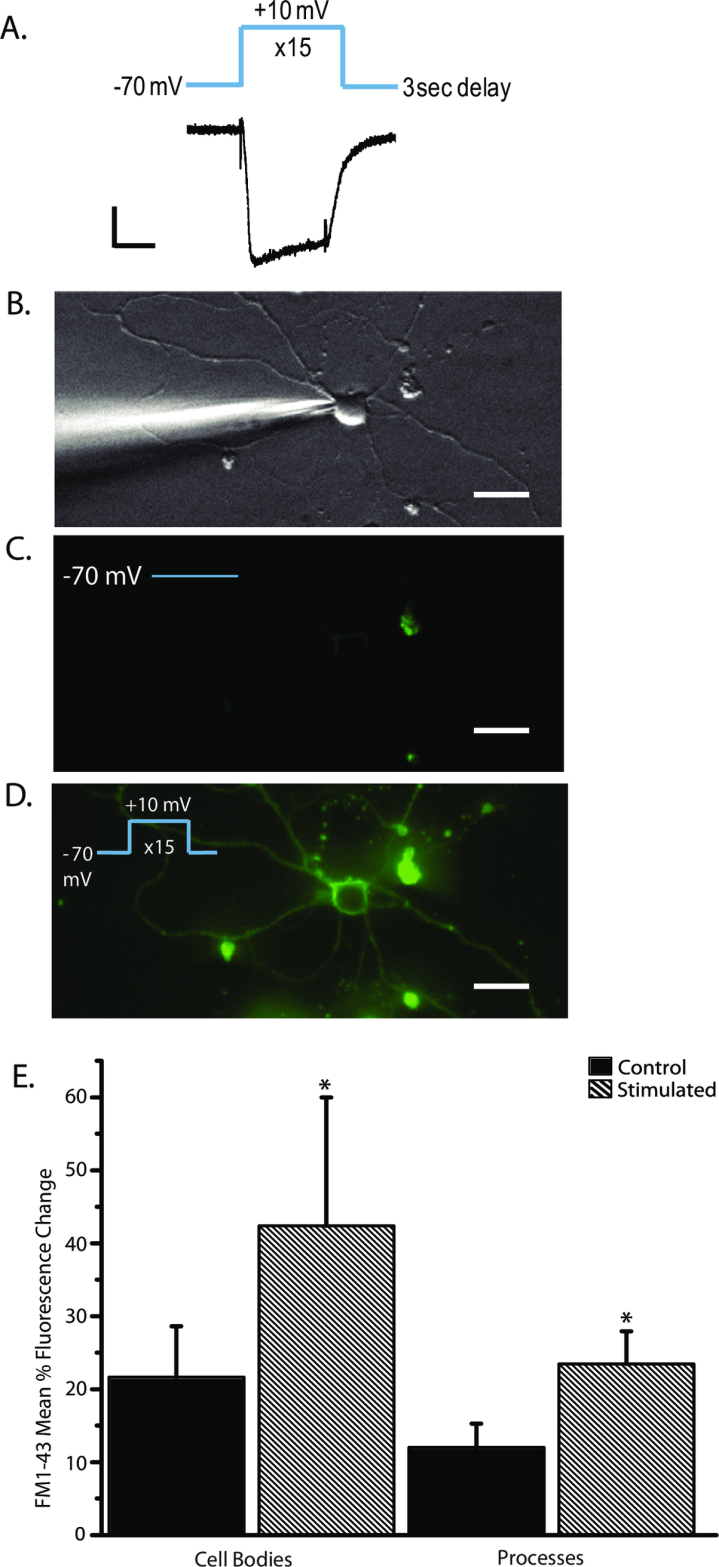
Voltage steps elicit endocytosis. **A**, Perforated-patch recording from a representative AC. The voltage was stepped from -70 mV to +10 mV for 100 msec and delivered 15 times with a 3 second delay between the steps. Currents resulting from all 15 voltage steps are shown. (Bottom) Scale bars are 100 pA and 50 ms. **B**, Another AC being recorded in the perforated patch configuration. **C**, Cells were exposed to the lipophillic dye FM1-43 (4 μM) for two minutes then imaged to establish a baseline control. **D**, An image after the series of voltage steps. Labelling can be seen in the cell body and in processes. Scale bars are 15 μm. **E**, Mean % fluorescence change ± SD. (paired t-test: cell bodies n = 6, p < .05; processes n = 8, p < .05).

**Fig 3:**
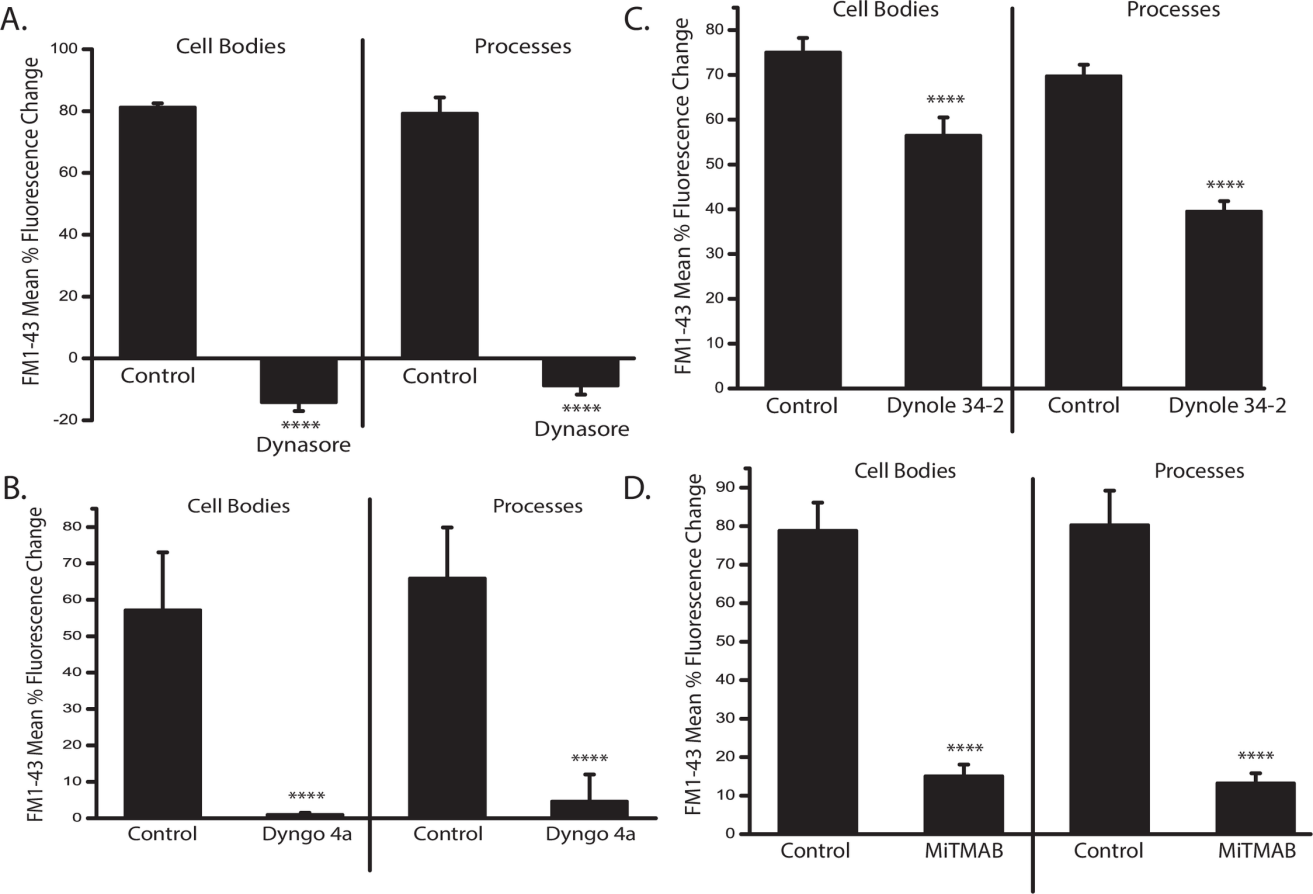
Dynamin inhibitors are effective in limiting activity-dependent FM1-43 labeling. **A-D,** Mean percent fluorescence change for cell bodies and processes for each of the dynamin inhibitors tested. Dynasore (but not the other inhibitors) increased baseline fluorescence such that after the voltage steps, the FM1-43 fluorescence was lower than control values giving rise to negative percent change in fluorescence. (Cell bodies: control n = 6; dynasore n = 6, p < 0.0001; Dyngo 4a n = 10, p < 0.0001; Dynole 34–2 n = 10, p < 0.0001; MiTMAB n = 6, p < 0.0001. Processes: control n = 6; dynasore n = 12, p < 0.0001, Dyngo 4a (30 μM), n = 12, p < 0.0001; Dynole 34–2 (10 μM) n = 10, p < 0.0001; MiTMAB (30 μM) n = 12, p < 0.0001, unpaired *t-*tests).

To test for the role of SVs in NOdrCl release, we sought to stimulate the synaptic vesicle cycle via depolarization under control conditions and in the presence of inhibitors of dynamin-dependent endocytosis. We hypothesized that inhibition of endocytosis under these conditions would preferentially deplete presynaptic vesicle pools and allow us to determine the contribution of SVs to NOdrCl. Synaptic vesicle exocytosis and subsequent endocytosis were achieved by activating voltage-gated Ca^2+^ channels. The voltage-gated Ca^2+^ channels expressed in cultured ACs that mediate neurotransmitter release are predominately L-type [43] and are susceptible to Ca^2+^-dependent inactivation [44]. Perforated-patch recordings were made to preserve the cytosolic environment for Ca^2+^-dependent exocytosis. We found that when 100 millisecond voltage steps from -70 mV to +10 mV were separated by 3 seconds, the effects of Ca^2+^-dependent inactivation was minimized (Fig 2A). To verify that the SV cycle was stimulated effectively, we delivered the voltage-steps in the presence of FM1-43, a tracker of endocytosis [45]. Increases in FM1-43 fluorescence in both cell bodies and processes were observed, indicating that the SV cycle was effectively activated and that the voltage steps promoted endocytosis throughout the cell (Fig 2B–2D). (Mean % fluorescence change in cell bodies: control, 21.9 ± 4.3, stimulated, 41 ± 10.9; processes: control, 11.4 ± 1.8, stimulated, 24.1 ± 4.2, Fig 3E).

### Dynamin inhibitors suppress activity-dependent FM1-43 labeling

To explore the role of SVs in the NOdrCl, the effects of dynasore [37, 46], Dyngo 4a [38], Dynole 34–2 [39] and MiTMAB [40, 47] were examined. The effectiveness of each of these pharmacological reagents as endocytic inhibitors was evaluated by testing the effects of each inhibitor on the voltage-dependent labeling with FM1-43. ACs were recorded in the perforated patch configuration. Control images were captured before the voltage steps, but after 2 min exposure to both FM1-43 and a dynamin inhibitor. Images were taken again after the voltage steps to +10 mV (15 steps, 100 ms) in the presence of either dynasore (80 μM), Dyngo 4a (30 μM), Dynole 34–2 (10 μM), or MiTMAB (30 μM). All of the inhibitors were effective in suppressing the accumulation of FM1-43 fluorescence following voltage steps (Fig 3A–3D) Dynasore cell bodies: control 81.3 ± 1.2%, stimulated -14.3 ± 2.7%; Dynasore processes 79.3 ± 5.1%, stimulated -8.9 ± 2.9%; Dyngo 4a cell bodies: control 57.2 ± 15.8%, stimulated 1.02 ± 0.5%; Dyngo 4a processes control 65.9 ± 13.9%, stimulated 4.7 ± 7.3%; Dynole 34–2 cell bodies: control 75.1 ± 3.2%, stimulated 56.5 ± 4.0%; Dynole 34–2 processes 69.8 ± 2.5%, stimulated 39.6 ± 2.2%; MiTMAB cell bodies: control 78.9 ± 7.2%, stimulated 15.1 ± 3.0%; MiTMAB processes control 80.3 ± 8.9%, stimulated 13.2 ± 2.6%). The control fluorescence images of the ACs exposed to dynasore were, on average, brighter before the voltage steps suggesting a higher level of constitutive endocytosis in dynasore and generating a net loss of fluorescence after the voltage steps (Fig 3A).

### Voltage steps alone can reduce the synaptic vesicle pool

**Fig 4:**
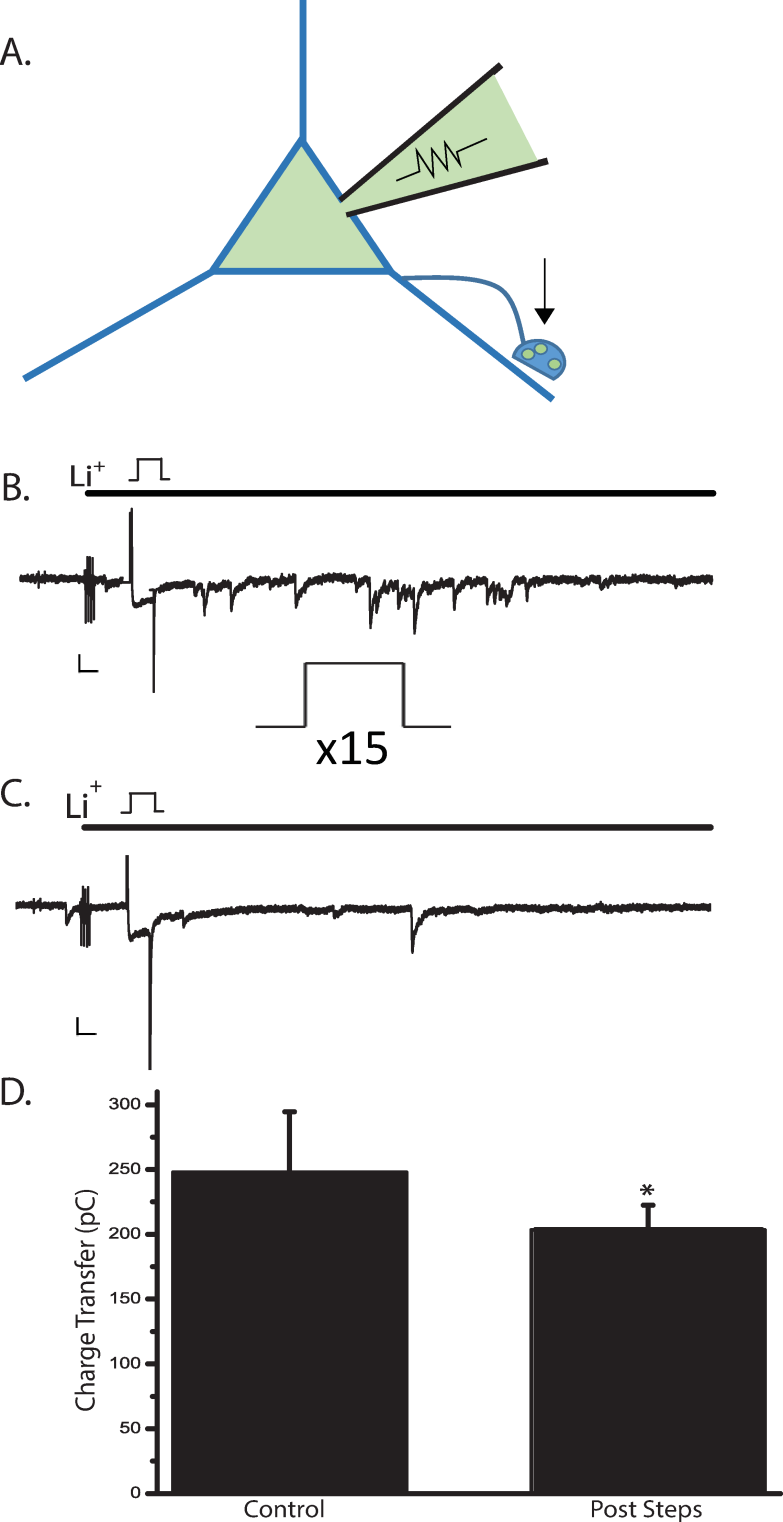
**Voltage steps alone can reduce the SV pool at autapses. A**, A depiction of an AC with an autaptic synapse. **B,** Autaptic synapses are activated by a single 100 msec voltage step from -70mV to +10mV in the perforated patch configuration. Outward autaptic currents during the voltage step to +10 mv are contaminated with inward voltage-gated Ca^2+^ currents. Contamination from the electrogenic inward Na/Ca exchange current after the voltage step is removed by replacing extracellular Na^+^ with Li^+^. The total time of exposure to Li^+^ is 4 seconds (black bar). After 15 voltage steps, the asynchronous phase of synaptic transmission is reduced **(C).** Scale bars are 25 pA and 100 ms. **D.** Mean charge transfer is plotted. (n = 7, p = .04, paired *t*-test).

From the FM1-43 experiments, we know that voltage steps (15 steps, -70 mV to +10 mV for 100 msec) were sufficient to stimulate exocytosis and subsequent endocytosis. To determine whether this same voltage step protocol was effective in depleting SVs in the absence of inhibition of endocytosis, we examined the postsynaptic responses of ACs before and after voltage steps alone. ACs in culture make synapses with other ACs. When isolated, they can also form autapses (Fig 4A). To examine whether repeated voltage steps can deplete SVs, we tested the autaptic currents before and after the voltage step protocol described in Fig 2A. Delivery of these voltage steps causes a reduction in the autaptic currents when compared to control (Fig 4B and 4C). During the voltage step to +10 mV, the inward voltage-gated Ca^2+^ currents and the outward synaptic currents contaminate each other, so synaptic currents were quantified from the end of the voltage step to the end of the record (Fig 4D). (Mean charge transfer (pC). Control 247.9 ± 46.5 pC; Post steps 203.3 ± 19.1 pC). These data indicate that the voltage steps alone can partially deplete SVs.

### Voltage steps are not sufficient block NOdrCl

**Fig 5:**
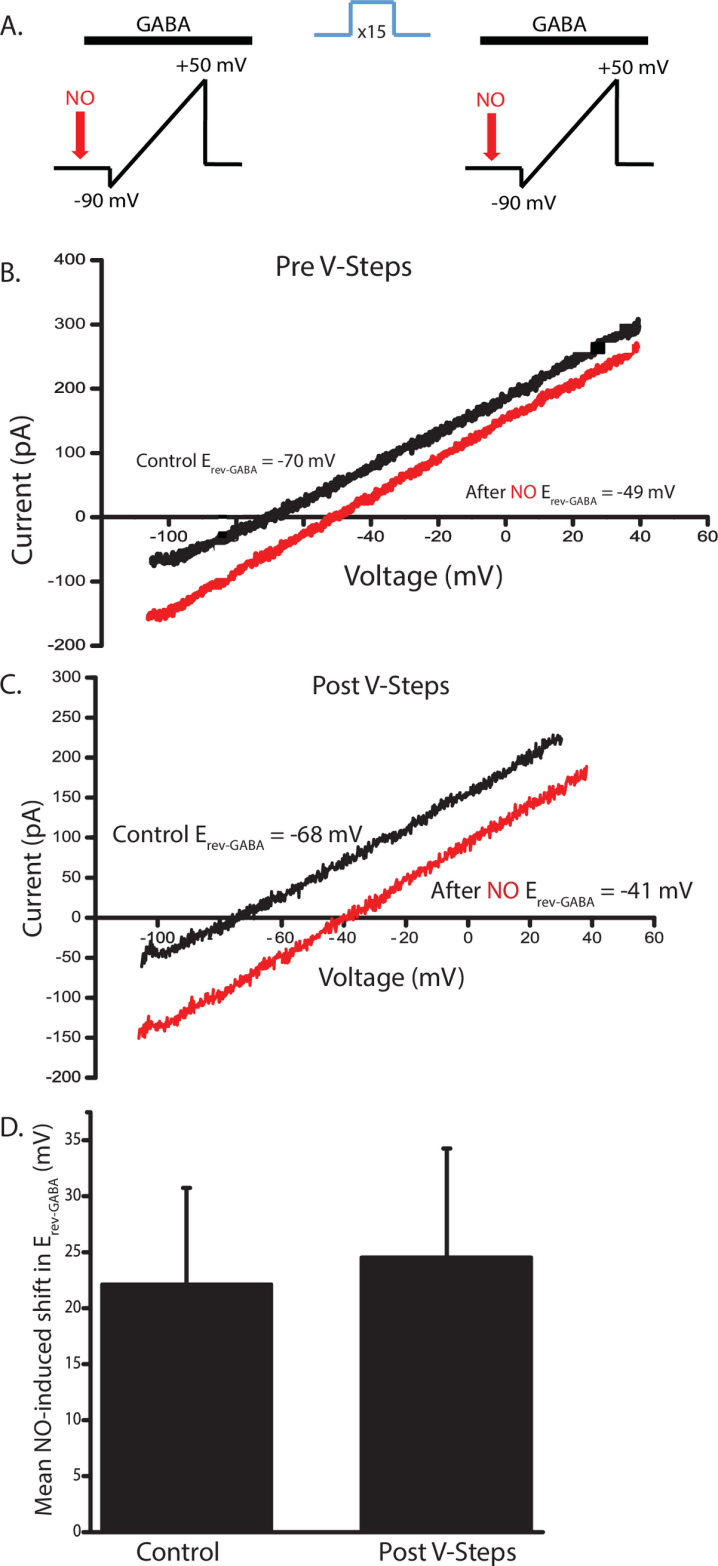
Voltage steps alone do not block NOdrCl. **A**, Schematic of the voltage ramp protocol. **B and C**, Currents elicited by voltage ramps in the presence of GABA are plotted from an AC recorded in the perforated patch configuration. Not shown are the ramp currents collected in the absence of GABA for the purpose of leak subtraction or the GABA-gated currents in response to voltage ramps collected prior to addition of NO. In this cell, the NOdrCl is larger after delivery of the voltage steps. **D**, On average, however, there was no significant difference in the amplitude of the shift before and after the voltage steps (n = 11, p = 0.6, paired t-test).

Because voltage steps can apparently stimulate enough exocytosis to reduce the SV pool, we tested whether voltage steps alone were sufficient to block NOdrCl. To evaluate the effects of the voltage step protocol on the NOdrCl, we combined a voltage ramp protocol to measure E_rev_-_GABA_ with the voltage-gated Ca^2+^ channel-activating voltage step protocol to initiate the synaptic vesicle cycle (Fig 5A). The voltage step protocol alone did not affect the NOdrCl. (Mean NO-induced shift (mV). Control 21.3 ± 6.1 mV; Post steps 24.7 ± 8.2 mV) (Fig 5B–5D). Taken together, these data suggest that although the voltage steps can reduce the SV pool, that alone is not sufficient to block the NOdrCl.

### Some dynamin inhibitors block the NOdrCl

**Fig 6:**
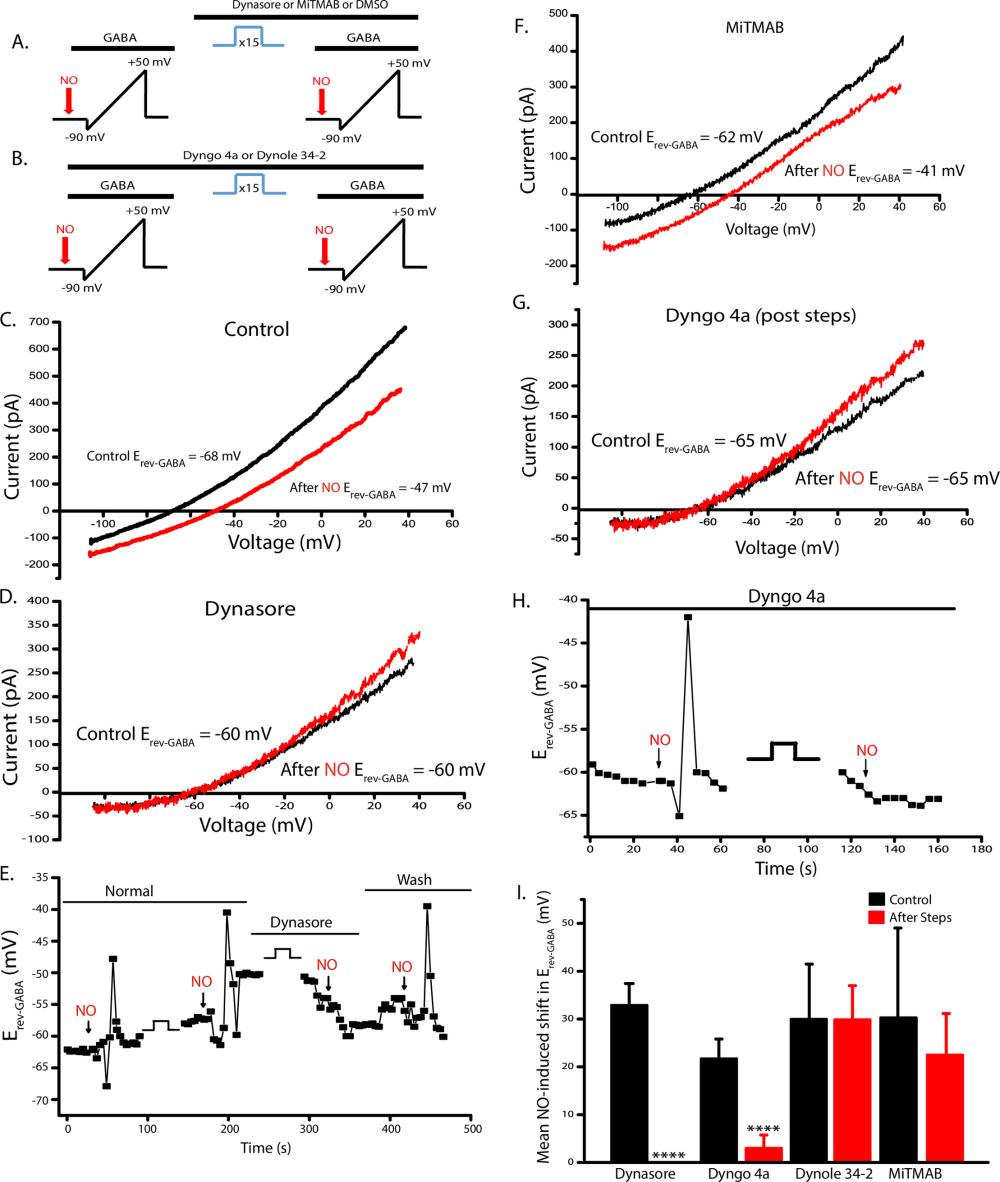
Dynasore and Dyngo-4a inhibit the NO-dependent shift in E_rev_-_GABA_. **A** and **B**, Schematics of the dynasore (80 μM) and MiTMAB (30 μM) (acute exposure) and the Dyngo 4a (30 μM) and Dynole 34–2 (10 μM) (20 min pre-incubation) experimental protocols, respectively. Not shown are the ramp currents collected in the absence of GABA for the purpose of leak subtraction or the GABA-gated currents in response to voltage ramps collected prior to addition of NO. **C.** Ramp currents recorded from an AC in the perforated patch configuration before and after (red trace) exposure to NO. **D.** Data from the same cell as in **C** after exposure to dynasore. **E.** E_rev_-_GABA_ for a different cell is plotted over time, and shows that the NO-dependent shift in E_rev-GABA_ can be reversed. **F.** GABA-gated currents in response to voltage ramps are shown for a representative AC exposed to MiTMAB reveal that this inhibitor does not block NO-dependent shift in E_rev_-_GABA_. **G.** Data plotted were recorded after the voltage step protocol and in the presence of Dyngo 4a. These data show that no shift occurred after NO application (red trace). **H.** E_GABA_ is plotted over the time course of the experiment for the same cell as shown in **G. I.** Quantified means ± SD the of NO-induced shifts of all inhibitors. (dynasore n = 12, p < 0.0001; Dyngo 4a n = 6, p < 0.0001; MiTMAB n = 5, p = 0.49; Dynole 34–2 n = 7, p = 0.89 unpaired *t*-tests).

To determine whether inhibition of endocytosis with dynamin inhibitors would affect the NOdrCl, GABA-gated ramp currents were measured before and after the voltage steps under control conditions and in the presence of one of the four inhibitors. Dynasore and MitMAB were applied acutely (Fig 6A). Under control conditions, the NOdrCl was observed (Fig 6C). The NOdrCl was also observed in control experiments when DMSO-alone was perfused onto cells (not shown, 30.1 ± 19.2 mV). However, the NOdrCl did not occur when the voltage steps and subsequent voltage ramps were delivered in the presence of dynasore (80 μM, Fig 6D, 6E and 6I). The effects of dynasore were partially reversible (Fig 6E, Mean NO-induced shifts in E_rev_-_GABA_, dynasore: control 32.9 ± 4.6 mV, dynasore post steps 0 ± 0 mV). When ACs were exposed to MiTMAB (30 μM) during the voltage steps and subsequent voltage ramps the NOdrCl persisted (Fig 6F and 6I) MiTMAB: control 30.3 ± 11.6 mV, MiTMAB post steps 29.9 ± 7.1 mV).

Dyngo 4a and Dynole 34–2 generally require pre-incubation due to their slower inhibitory effects on GTPase activity [38, 40]. For these experiments ACs were exposed to Dyngo 4a (30 μM) or Dynole 34–2 (10 μM), for 20 minutes prior to recording and the reagents remained throughout the recording (Fig 6B). In the presence of Dyngo-4a, but before the voltage steps, injecting NO caused a transient shift in the reversal potential of the GABA-gated current (Fig 6H). After the voltage steps, however, Dyngo 4a blocked the NOdrCl (Fig 6G, 6H and 6I, Dyngo 4a: control 21.7 ± 4.0 mV, Dyngo 4a post steps 3.0 ± 2.7 mV). This dependence upon the voltage steps, indicates that Ca^2+^-dependent exocytosis was required along with the inhibitor. Dynole 34–2 was ineffective in inhibiting the shift in E_rev_-_GABA_ (Fig 6I, Dynole 34–2 control 30.9 ± 19.1 mV; Dynole 34–2 post steps 30.0 ± 11.2 mV).

### Dynamin inhibitors affect voltage-dependent Ca^2+^ signaling

Although dynasore, Dyngo 4a and Dynole 34–2 are all known to inhibit dynamin’s GTPase activity, only dynasore and Dyngo 4a inhibited the NOdrCl. Off-target effects have been demonstrated for dynasore [48]. It has been shown that dyngo 4a and Dynole 34–2 are more potent and less cytotoxic than dynasore, however, off-target effects of Dyngo 4a & Dynole 34–2 are not yet known. There is evidence, however, that MiTMAB can inhibit voltage-gated Ca^2+^ channels required for exocytosis in rat adrenal chromaffin cells [47].

**Fig 7:**
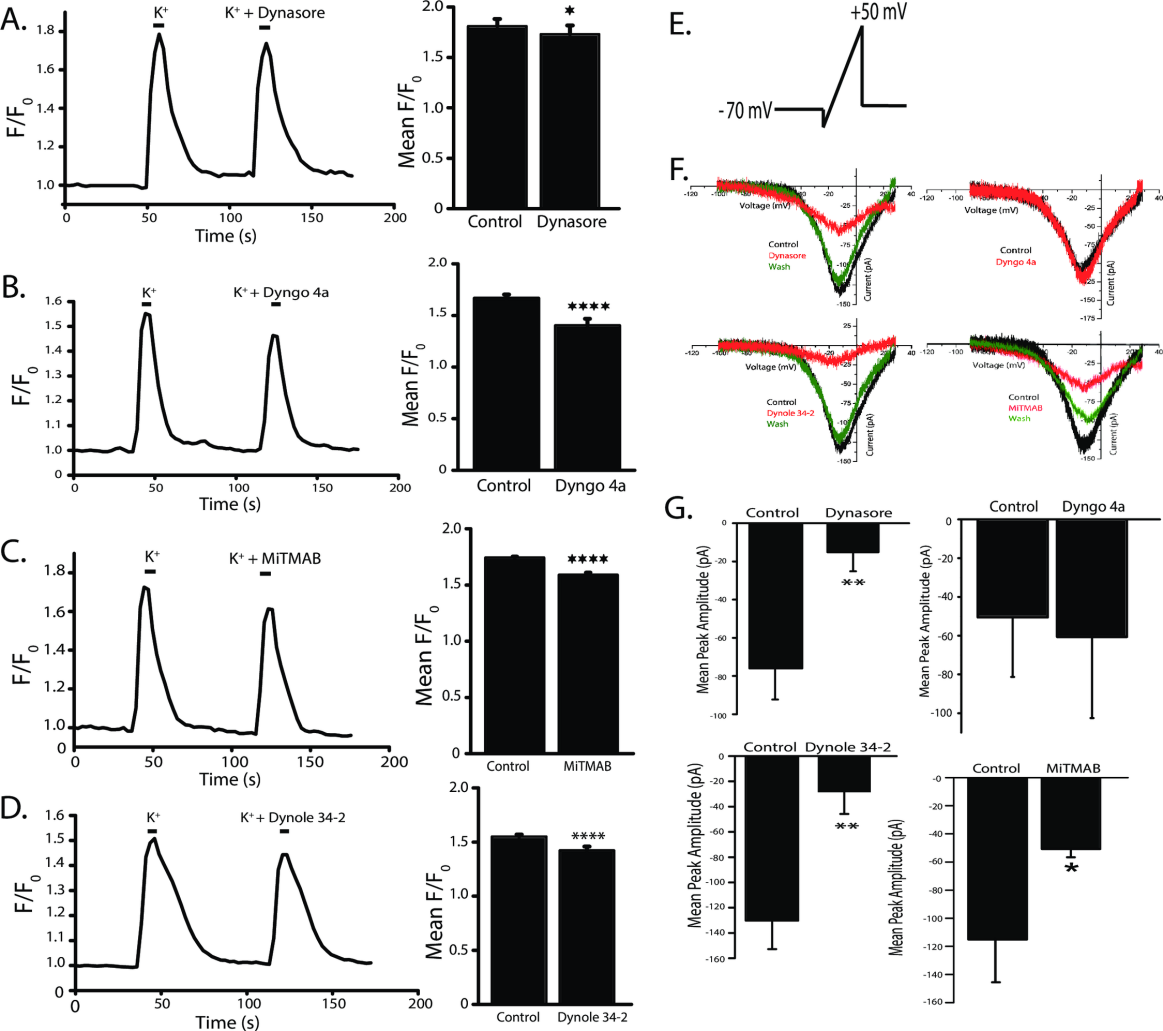
Dynamin inhibitors interfere with voltage-dependent Ca^2+^ signaling and voltage-gated Ca^2+^ currents. **A-D,** (Left) Ca^2+^ imaging data from OGB 488-loaded (1 μM) ACs. A 50 mM K^+^ -induced depolarization causes an increase in OGB fluorescence, which indicates an increase in [Ca^2+^]. Acute exposure of dynasore, Dyngo 4a, Dynole 34–2 and MiTMAB all reduce the voltage-dependent Ca^2+^ increases. Mean quantified F/F_0_ ± SD. Data collected from regions of interest (ROIs) in cell bodies. (Control and dynasore, n = 44; p < 0.05; Control and Dyngo n = 56; p < 0.0001; Control and MiTMAB n = 44, p< 0.0001; control and Dynole 34–2, n = 63, p < 0.0001, paired *t*-tests). **E.** Schematic of the voltage ramp protocol used to elicit voltage-gated Ca^2+^ currents. Cells in the ruptured patch configuration were voltage-clamped at -70 mV for 500 ms. Then the voltage was stepped briefly down to -90 mV, ramped to +50 mV, and then back down to -70 mV. **F**, Representative voltage-clamp recordings from four representative ACs. Dynasore, Dynole 34–2, and MiTMAB reduce the peak amplitude of the Ca^2+^ currents and the effects were at least partially reversible. **G**, Mean peak amplitudes are plotted for four populations of cells (dynasore n = 6, p<0.01; MiTMAB n = 5, p<0.01; Dynole 34–2 n = 5, p<0.01; Dyngo 4a n = 5, p = 0.5, paired *t*-tests).

To address the possibility that the dynamin inhibitors were affecting some aspect of Ca^2+^ signaling, we performed imaging experiments on ACs loaded with the Ca^2+^ indicator Oregon Green 488 BAPTA-1 (OGB) to investigate the effects of all the dynamin inhibitors on voltage-dependent Ca^2+^ increases. In these experiments, a high K^+^ solution (50 mM) was perfused into the culture dishes for 6 seconds to depolarize the cells and activate voltage-gated Ca^2+^ channels. Dyngo 4a, Dynole 34–2, dynasore, and MiTMAB were applied in addition to the high K^+^ depolarization, and all reduced the OGB fluorescence increase (Fig 7A–7D). (Mean F/F_0_. Control for dynasore: 1.80 ± 0.04, dynasore 1.73 ± 0.08; Control for Dyngo 4a 1.7 ± 0.03, Dyngo 4a 1.4 ± 0.1; Control for MiTMAB 1.75 ± 0.01, MiTMAB 1.59 ± 0.02; Control for Dynole 34–2 1.55 ± 0.04, Dynole 34–2 1.42 ± 0.03). Although Dyngo 4a and Dynole 34–2 were applied acutely in these cells, we did pre-incubate separate cells in the inhibitors (as in Fig 6) and also saw a reduction in the OGB fluorescence when compared to untreated control cells (Data not shown, Mean F/F_0_ ± SD. Control for Dyngo: 1.3 ± 0.05; Dyngo 4a 1.2 ± 0.02; control for Dynole 34–2: 1.55 ± 0.03; Dynole 34–2 1.04 ± 0.02). In some cells, the effect of the inhibitors in high K^+^ were tested before the control depolarization in order to detect any inhibitor-independent effects on the Ca^+2^ signal amplitude over time. No such effects were observed.

In ACs, voltage-gated L-type Ca^2+^ channel activity initiates neurotransmitter release [43, 49, 50]. To examine whether these dynamin inhibitors affect L-type Ca^2+^ channel currents, we used voltage ramps to elicit voltage-gated Ca^2+^ current (Fig 7E and 7F. Because the amplitude of voltage-gated Ca^2+^ currents are variable in these cells, we only tested the acute effects of the inhibitors to allow for “within cell” comparisons. Dynasore, Dynole 34–2 and MiTMAB significantly reduced the peak amplitude of the Ca^2+^ current (Mean peak amplitude (pA). Dynasore control -76.0 ± 16.2 pA, dynasore -11.2 ± 20.4 pA; Dyngo 4a control -50.5 ± 30.8 pA, Dyngo 4a -60.0 ± 41.9 pA; Dynole 34–2 control -130.3 ± 22.6 pA, Dynole 34–2–31.6 ± 6.3 pA; MiTMAB control -118.1 ± 19.7 pA. MiTMAB -52.2 ± 2.3 pA) (Fig 7G). The inhibitors did not affect the voltage-dependence of the Ca^2+^ current. These data demonstrate that the Ca^2+^ channels expressed by ACs can be inhibited by some dynamin inhibitors. We observed that the effects on Ca^2+^ currents were more dramatic than the effects on voltage-dependent Ca^2+^ elevations, suggesting that Ca^2+^ release from stores plays a substantial role in the Ca^2+^ elevations.

### Actin disrupters do not affect the NOdrCl

**Fig 8:**
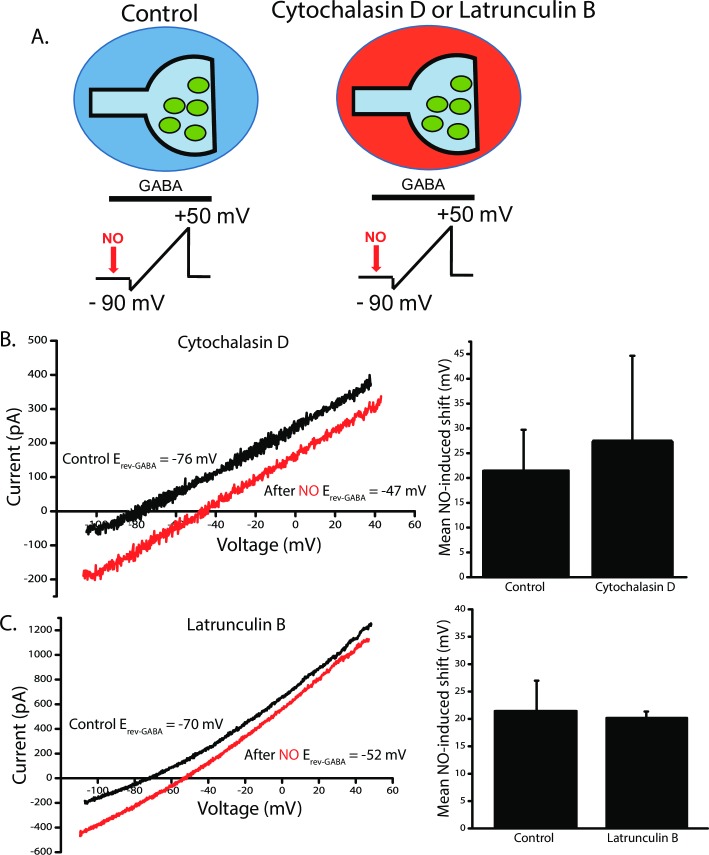
**Dynamin inhibitors do not block the NOdrCl via disruption of actin. A**, Cartoon of experimental setup. In control cells, voltage ramps were delivered in the perforated patch configuration before and after injections of NO in normal external solution. Other ACs were pre-treated with either Cytochalasin D (4 μM) or Latrunculin B (4 μM) for at least 45 minutes. **B** and **C,** left. Leak-subtracted GABA-gated currents reveal a shift in E_rev_-_GABA_ after NO (red trace) in ACs recorded in either cytochalasin D (B) or latrunculin B (C). Mean NO-induced shifts ± SD. (Cytochalasin D, n = 7, p = 0.41, Latrunculin B, n = 6, p = 0.43, unpaired *t*-tests).

In addition to its role in endocytic fission, dynamin interacts with and regulates actin filaments, [51, 52] which has the potential to regulate numerous other cellular functions [53]. Interestingly, F-actin has been shown to directly interact with CFTR [54, 55] and to regulate its function [56]. Thus, a dynasore-mediated disruption in actin filament regulation might also affect the function of CFTR, a key player in NOdrCl. To determine whether disruption of actin regulation underlies the inhibition of NOdrCl by dynamin inhibitors, we employed two actin polymerization disruptors Cytochalasin D [57, 58] and Latrunculin B [59]. ACs were pre-incubated in either Cytochalasin (4μM) or Latrunculin B (4μM) for 45 minutes (Fig 8A) and the inhibitors were present during the recordings. NO-dependent shifts in E_rev_-_GABA_ persisted in cells pre-treated with the inhibitors. When compared to control cells recorded on the same day, there were no significant difference in the shift amplitudes for ACs recorded in the presence of the inhibitors (Mean NO-induced shift (mV). cytochalasin control 21.5 ± 8.3 mV, cytochalasin D 27.5 ± 17.1 mV; latrunculin B control 21.4 ± 4.2 mV, latrunculin B 20.2 ± 1.1 mV) (Fig 8B and 8C). These data suggest that dynasore and Dyngo 4a are not inhibiting the NOdrCl by altering dynamin’s interaction with actin.

### GTP-γS does not affect the NOdrCl

**Fig 9:**
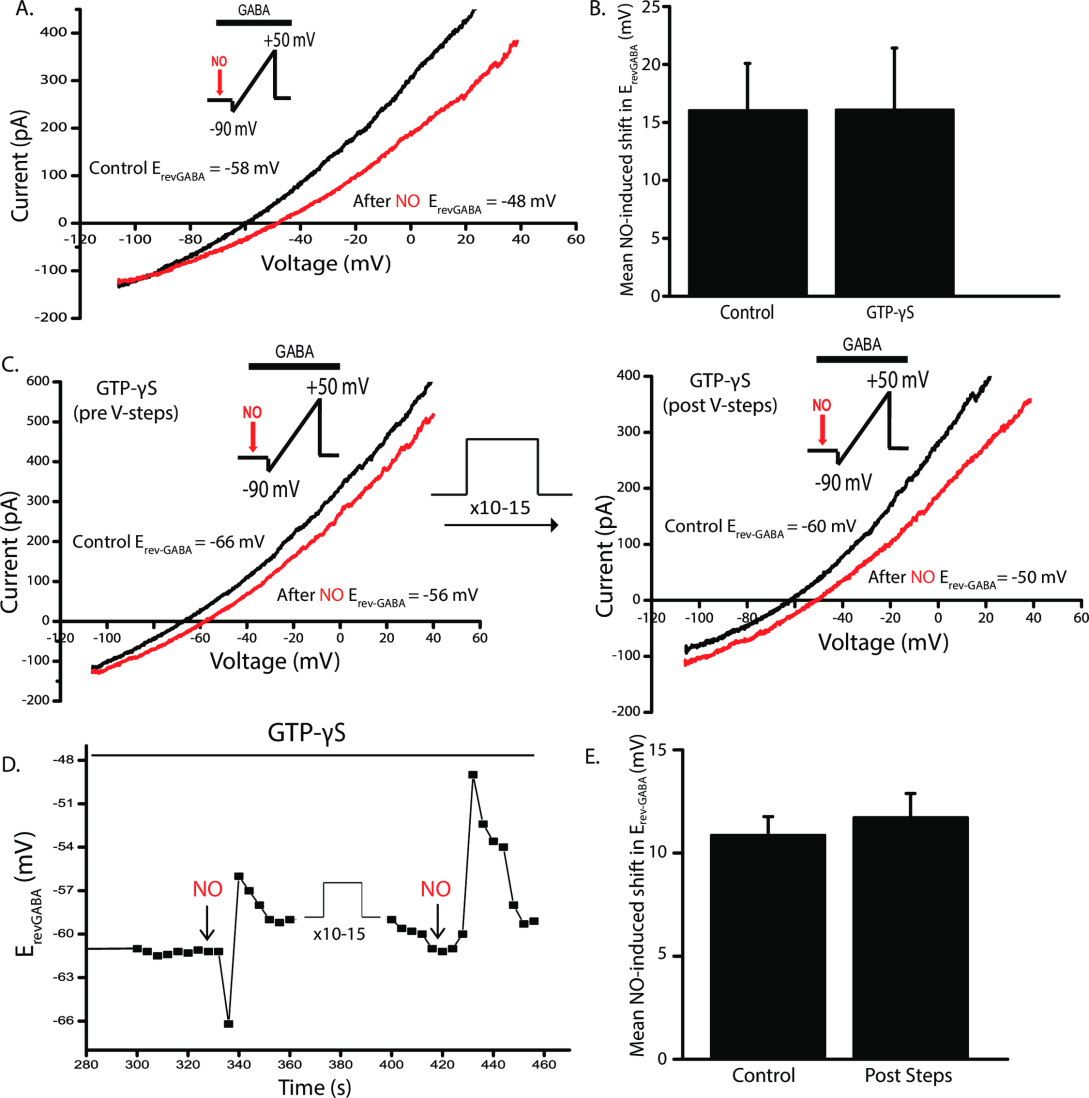
GTP-γS does not mimic the effects of dynasore and Dyngo 4a on the NOdrCl. **A.** In the ruptured patch configuration, GTP-γS (4 mM) replaced GTP in the ATP regeneration system to function as a general GTPase inhibitor. About 4 minutes after gaining access to the cell, voltage ramps were delivered before and after injections of NO and the NO-dependent shift in E_rev_-_GABA_ was still observed. **B.** Mean NO-induced shifts ± SD (Unpaired *t*-test: control n = 7, GTP-γS n = 7, p = 0.98). **C.** In separate cells, voltage ramps were delivered before and after injections of NO 4 minutes after rupturing the patch. Then voltage steps were delivered to stimulate exo- and endocytosis, followed by another set of voltage ramps and NO. The NO-dependent shift in E_rev_-_GABA_ remained. **D.** E_rev_-_GABA_ is plotted over the time course of the experiment for the cell shown in **C. E.** Mean NO-induced shifts ± SD. (n = 3, p = 0.4, paired *t*-test).

If dynasore and Dyngo-4a block the NOdrCl by interfering with the GTPase function of dynamin, then we might be able to mimic the effect with a general inhibitor of GTPase function. GTP-γS is a non-hydrolysable analogue of GTP that can function as a GTPase inhibitor [60]. GTP-γS (4mM) replaced GTP in the ATP regeneration system of the internal solution. With GTP-γS in the pipet, ruptured-patch recordings were only viable for up to about 5 minutes. After gaining access to the cell with GTP-γS in the pipet, the voltage ramp protocol shown in Fig 6A was delivered and the NO-dependent shift in E_rev_-_GABA_ was observed. This representative trace was collected about 4 minutes after gaining access to the cell. (Fig 9A and 9B) ((Mean NO-induced shifts ± SD. Control: 16 ± 4.0 mV. GTP-γS: 16.1 ± 5.3 mV, unpaired *t*-test).

The effects of GTP-γS were also tested under conditions of active exocytosis and endocytosis. Voltage ramps in the presence of GABA were delivered before and after injections of NO, followed by the voltage step protocol previously described, and then the ability of NO to shift E_rev_-_GABA_ was re-tested. At least on this time frame (about 5 minutes total time since gaining electrical access to the cell), GTP-γS did not block the NO-dependent shift in E_rev_-_GABA_. (Fig 9C–9E) (Mean NO-induced shift ± SD. Control: 10.2 ± 0.9 mV. Post Steps 11.7 ± 1.2 mV). Recordings were also made in the ruptured patch configuration using a GTP-free internal solution and the NOdrCl was not blocked (data not shown, 20.1 ± 2.5 mV). Due to the limited duration of the recordings in these experiments, the possibility remains that GTP-γS may have incompletely filled the neuronal processes. These data, however, do raise the possibility that dynasore and Dyngo-4a, both of which are thought to function via GTPase inhibition, are inhibiting endocytosis and the NOdrCl by some other mechanism.

### Dynasore affects neurotransmitter release from ACs

**Fig 10:**
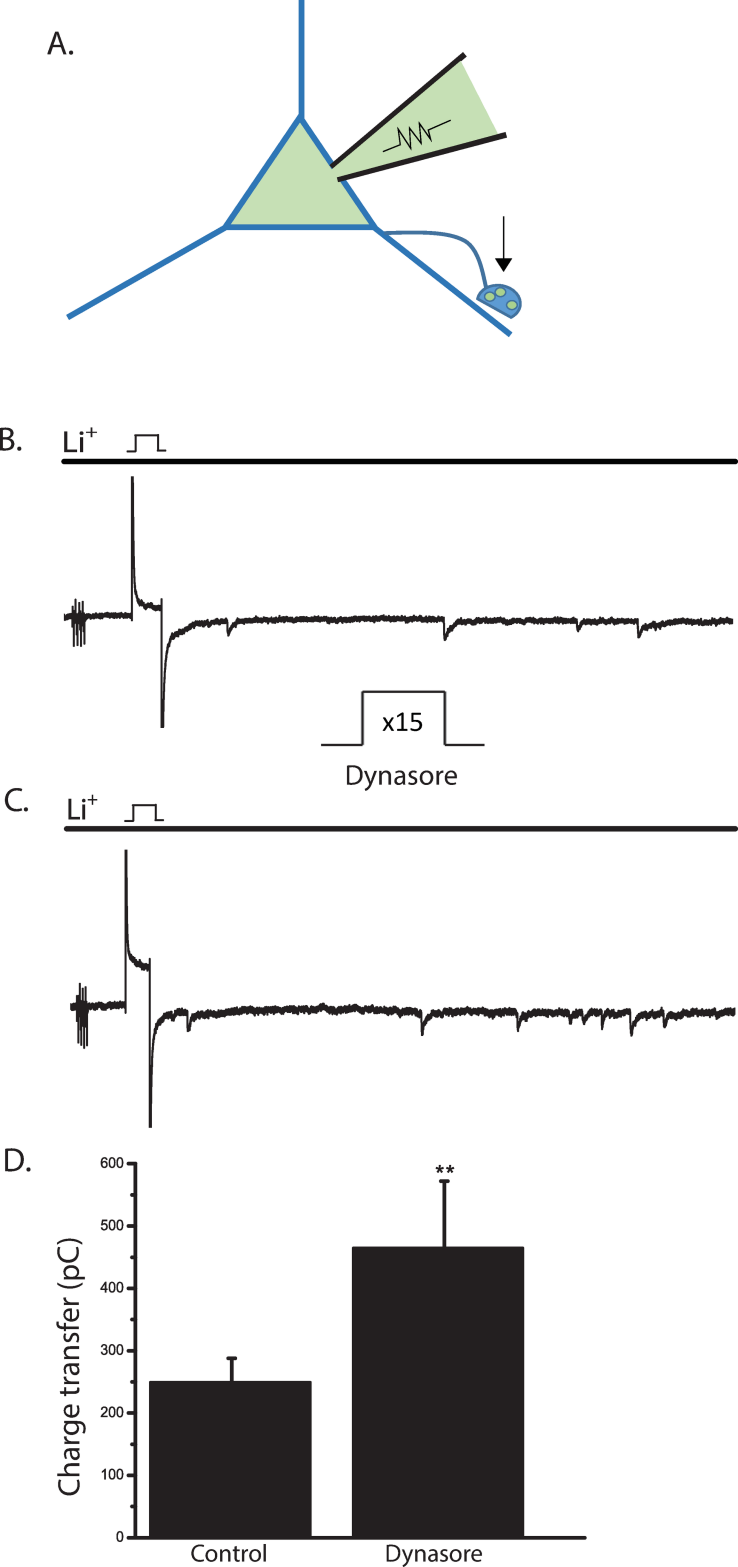
Dynasore promotes neurotransmitter release at amacrine cell autapses. **A,** A depiction of an AC in with an autaptic synapse. **B,** Autaptic synapses are activated by a single 100 msec voltage step from -70 mV to +10 mV recorded in the perforated patch configuration. Autaptic currents during the voltage step are contaminated with the voltage-gated Ca^2+^ currents. Contamination from the electrogenic inward Na/Ca exchange current after the voltage step is removed by replacing extracellular Na^+^ with Li^+^. The total time of exposure to Li^+^ is 4 seconds (black bar). **C,** After 15 voltage steps in the presence of dynasore, autaptic currents reveal enhanced neurotransmitter release. **D,** Mean charge transfer ± SD. (n = 6, p = .0063, paired *t*-test).

Dynasore has been shown to increase the probability of evoked transmitter release at the frog NMJ and to increase spontaneous synaptic event frequency in the nucleus of the solitary tract (NTS) neurons [61, 62]. If dynasore similarly affects presynaptic function in ACs, then this might contribute to the inhibitory effect of dynasore on the NOdrCl. To determine whether AC synaptic exocytosis was altered by dynasore, we tested its effects on autaptic currents (Fig 10A–10C). Dynasore increased the asynchronous postsynaptic currents (Fig 10D). (Mean charge transfer ± SD. Control 245.1 ± 35.9 pC; dynasore post steps 464.9 ± 107.2 pC) indicating that dynasore promotes synaptic exocytosis from ACs.

### Pitstop 2 inhibits the NOdrCl

**Fig 11:**
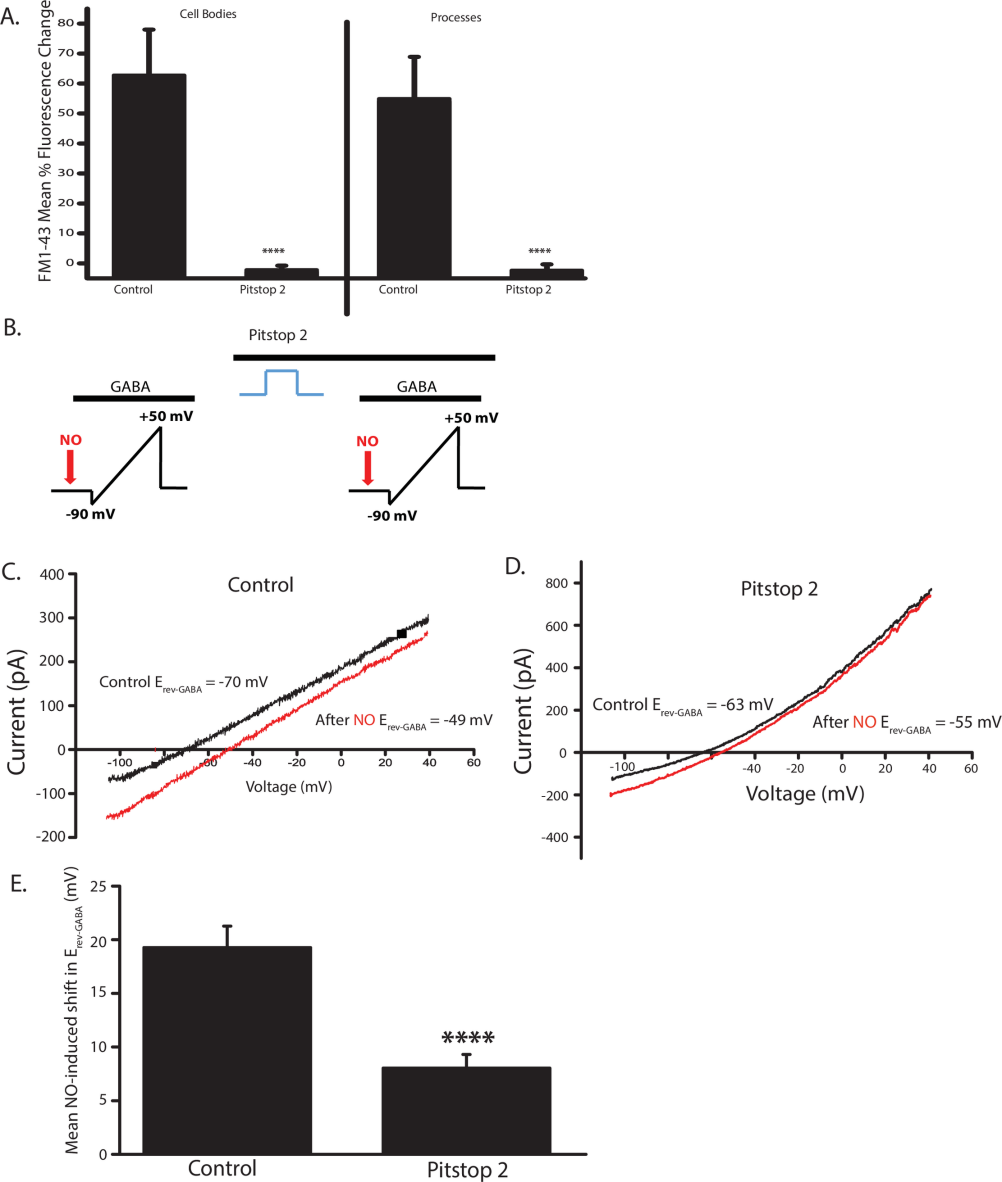
Pitstop 2 reduces the NO-dependent shift in E_GABA_. **A.** Mean percent fluorescence change for cell bodies and processes in the presence of Pitstop 2 (20 μm). The clathrin inhibitor reduces voltage-dependent FM1-43 fluroescence increases, indicating that it is an effective SV inhibitor (cell bodies, n = 9, p<0.0001; processes, n = 18, p<0.0001, unpaired t-test). **B.** Schematic of voltage ramp experiment. ACs were recorded in the perforated patch configuration. **C.** and **D.**Representative trace of E_GABA_ measurements before and after voltage steps in the presence of Pitstop 2. **E.** Mean NO-induced shifts ± SD. Pitstop 2 reduces the NOdrCl. (n = 7, p<0.0001, paired *t*-tests).

The effects of dynasore and Dyngo 4a on the NOdrCl are consistent with a role for SVs as a releasable source of Cl^-^. However, the additional effects of these compounds that we describe here as well as the inability of GTPγS to mimic the effects of dynasore and Dyngo 4a make a conclusion difficult to draw. Therefore, we sought to inhibit endocytosis by an alternative mechanism. Pitstop 2 has been thought to inhibit the interactions between clathrin and adapter proteins that are required for clathrin coat assembly [63] although it has been demonstrated to inhibit clathrin-independent endocytosis as well [64]. To determine whether Pitstop 2 was effective in inhibiting endocytosis, we combined this reagent with the voltage and imaging protocol used in Figs 2 and 3. Pitstop 2 inhibited voltage-dependent FM1-43 fluorescence increases (Cell bodies control 61.5 ± 16.6%, Pitstop 2 1.1 ± 0.05%; processes control 54.9 ± 15.0%, Pitstop 2 1.1 ± 0.03%,) (Fig 11A). The effectiveness of Pitstop 2 in inhibiting the NOdrCl was tested by repeating the experiment shown in Fig 5A but delivering the voltage steps in the presence of Pitstop 2 rather than the dynamin inhibitors (Fig 11B). Pitstop 2 inhibited the NOdrCl by about 50% (Mean NO-induced shift in E_GABA_ ± SD. Control: 19.3 ± 2.0 mV; Pitstop 2: 8.1 ± 1.3 mV) (Fig 11C–11E). The ability of another endocytosis inhibitor that functions by a completely different mechanism suggests that depletion of the SV pool reduces the sources of Cl^-^ during NOdrCl.

## Discussion

We embarked on these experiments to determine whether SVs contribute to the cytosolic Cl^-^ elevations generated by NO. Our results demonstrate that dynamin inhibitors can block endocytosis as measured by FM1-43 labeling and two out of the four reduced or blocked the NOdrCl. We also found that dynamin inhibitors can affect Ca^2+^ signaling, voltage-gated Ca^2+^ currents, and synaptic transmission. Nonetheless, the clathrin inhibitor Pitstop 2 also reduced the NOdrCl, suggesting that the disruption of SV endocytosis is one factor involved in the inhibition of the NOdrCl by dynasore and Dyngo 4a.

### Variable effects of dynamin inhibitors

Although each of the dynamin inhibitors were demonstrated to inhibit endocytosis as measured by FM1-43 labeling, Dynole 34–2 and MiTMAB were the least effective. Similarly, dynasore and Dynole 4a were effective in blocking the NOdrCl whereas Dynole 34–2 and MiTMAB were not. It is possible that it is due to the particular isoform of dynamin that is being inhibited, however, dynasore, Dynole 34–2 and MiTMAB are effective on all three mammalian isoforms of dynamin with only Dyngo-4a showing a preference for dynamin 1 [27, 65]. Another possibility is that the differences in effectiveness are due to species differences although the high level of identity (>90%) between chicken and rodent dynamins makes this explanation less likely. It has recently been demonstrated that at the neuromuscular junction, dynasore and MiTMAB have distinctive actions on endocytosis where dynasore inhibited only an early phase of endocytosis and MiTMAB inhibited only a late phase [66]. The stimulus conditions in those experiments were much different than ours with exocytosis stimulated by 1000 action potentials delivered at different frequencies. Our presynaptic stimulation involved 15 100 msec voltage steps providing a total duration of depolarization of 1.5 sec whereas the duration of action potential stimulation in Linares-Clemente et al. (2015) ranged from ~10–100 sec. Nonetheless, their experiments reveal that MiTMAB might be an efficient inhibitor of dynamin only under a specific set of circumstances. Although Dynole 34–2 and MiTMAB were less effective in inhibiting activity-dependent FM1-43, they still had significant effects. The lack of effect on the NOdrCl suggests that the effect on the NOdrCl may have some sort of threshold component.

### Additional effects of dynamin inhibitors in ACs

Here, we provide evidence that dynamin inhibitors can alter Ca^2+^ signaling and synaptic transmission in ACs. What is not clear is whether these are “off-target” effects in the sense that they are not due to inhibition of dynamin function or are these effects due to inhibition of dynamin functions that are thought to be unrelated to endocytosis? A well-established additional function of dynamin is regulation of the actin cytoskeleton [51]. However, the other effects of dynasore that we demonstrate might be due the actin-regulating role of dynamin. A functional link between dynamin, the actin cytoskeleton and L-type Ca^2+^ channel activity has been demonstrated for αT3-1 gonadotroph cells [67]. Signaling via activation of the gonadotrophin releasing hormone receptor typically leads to cytoskeletal rearrangement which they show is required for normal activation of L-type Ca^2+^ channels in these cells. These results raise the possibility that dynasore and Dynole 34–2 might inhibit L-type activity in ACs via dysregulation of the actin cytoskeleton. Regardless of the mechanism, inhibition of L-type Ca^2+^ channels does not explain the effects of dynasore and Dyngo 4a on the NOdrCl. First, Dyngo 4a inhibited the NOdrCl, but did not inhibit voltage-dependent Ca^2+^ currents. Additionally, inhibition of L-type channels would minimize our ability to deplete synaptic vesicles so would likely lessen the effects of the inhibitors on the NOdrCl.

We also show that dynasore increased evoked synaptic transmission at AC autapses. Although dynasore has been shown to promote neurotransmitter release from the frog NMJ [61] and in rodent nucleus of the solitary tract neurons [62], the effect there was to increase spontaneous neurotransmitter release rather than evoked. At the neuromuscular junction the effect was partially Ca^2+^-dependent and it was revealed that minutes-long exposure to dynasore also elevated basal Ca^2+^ levels in terminals. In ACs, no Ca^2+^ elevations were observed in response to exposure to dynasore or any of the other dynamin inhibitors arguing against a Ca^2+^-dependent effect, especially in light of the inhibitory effect of dynasore, Dynole 34–2, and MiTMAB on AC Ca^2+^ current amplitude.

### Pitstop 2

Pitstop 2 was developed by screening compounds for interference between the terminal domains and known binding partners of clathrin such as amphiphysin and AP180 *in vitro* [63]. Since its development, Pitstop 2 has been used in a wide variety of preparations and has been demonstrated to be an effective inhibitor of endocytosis, however, its mechanism of action *in vivo* is a matter of debate [2]. It has been shown to inhibit both clathrin-dependent and independent endocytosis [64, 68] which calls into question its predicted interactions with clathrin. These and other observations have led to suggestions that Pitstop 2 actually interferes with a step downstream from coat formation [2]. In hippocampal neurons Pitstop 2 was also reported to affect vesicle acidification and mitochondrial pH, however these data were not shown and the degree and nature of the alterations were not stated, making it difficult to evaluate the importance of these potential effects in ACs [69]. Given these caveats, however, the consistency of the effects of dynasore, Dyngo 4a and Pitstop 2 in inhibition of the NOdrCl suggest that fully functional endocytosis is required for NO to elevate cytosolic Cl^-^.

### Significance

Here, we report that NO, likely in the range of 100’s of nanomolar, initiates release of Cl^-^ and that this release is dependent upon endocytosis. What is the evidence that these conditions actually occur in the retina? Imaging experiments using 4,5-diaminofluoroscein (DAF) to detect NO production in slices of turtle retina demonstrate light-dependent DAF signals are generated in subsets of cells throughout the retina [70]. Interestingly, DAF signals were largely confined to the boundaries of the cell bodies and processes in ACs, whereas cells in the ganglion cell layers had DAF signals that extended several microns away from the cell body. NO microelectrode measurements made just outside a DAF-positive cell in the ganglion cell layer detected NMDA-stimulated NO concentrations of about 200 nM [70]. Presumably, the concentration in the cytosol is higher. Furthermore, light stimulation of the retina within the physiological range results in S-nitrosylation patterns that are intensity-dependent [71]. This is significant because S-nitrosylation occurs over higher concentration ranges than does NO-dependent activation of soluble guanylate cyclase which can occur in the low nanomolar to picomolar range [72, 73]. Importantly, we have previously demonstrated that the NOdrCl operates independent of soluble guanylate cyclase [1].

The involvement of SVs in the NOdrCl is especially relevant to AC function because ACs participate in serial [74] and reciprocal synapses [21] such that presynaptic Cl^-^ can affect the sign of nearby incoming GABAergic synapses in a highly localized fashion [22–24]. We have also recently demonstrated that the NO donor SNAP can increase spontaneous and evoked GABA release via presynaptic activation of TRPC5 channels [41, 75] providing an additional NO-dependent mechanism for enhancing GABAergic output from ACs.

The impact of NO as an enhancer of GABAergic output in cultured ACs supports the hypothesis that NO can increase the GABAergic output onto ganglion cells in the retina. This effect of NO had been demonstrated in dark-adapted ferret retina where ON and OFF light responses were recorded in ganglion cells [76]. NO suppressed the light-dependent activity in both ON and OFF ganglion cells but the effect was especially strong in the OFF pathway. Furthermore, this effect was thought to be due to an increase in inhibitory output from amacrine cells. Our results in ACs also have implications for NO effects on GABAergic synapses elsewhere in the brain because we have demonstrated that hippocampal neurons also have an NO-releasable store [1]. Understanding the mechanisms underlying the NO-dependent switch from inhibitory to excitatory input will make an important contribution to our understanding of the flexibility of synaptic transmission in the CNS.

## Supporting information

S1 Appendix Complete data list. (XLSX)
